# Directly converting CO_2_ into a gasoline fuel

**DOI:** 10.1038/ncomms15174

**Published:** 2017-05-02

**Authors:** Jian Wei, Qingjie Ge, Ruwei Yao, Zhiyong Wen, Chuanyan Fang, Lisheng Guo, Hengyong Xu, Jian Sun

**Affiliations:** 1 Dalian National Laboratory for Clean Energy, Dalian Institute of Chemical Physics, Chinese Academy of Sciences, Dalian 116023, China; 2 University of Chinese Academy of Sciences, Beijing 100049, China

## Abstract

The direct production of liquid fuels from CO_2_ hydrogenation has attracted enormous interest for its significant roles in mitigating CO_2_ emissions and reducing dependence on petrochemicals. Here we report a highly efficient, stable and multifunctional Na–Fe_3_O_4_/HZSM-5 catalyst, which can directly convert CO_2_ to gasoline-range (C_5_–C_11_) hydrocarbons with selectivity up to 78% of all hydrocarbons while only 4% methane at a CO_2_ conversion of 22% under industrial relevant conditions. It is achieved by a multifunctional catalyst providing three types of active sites (Fe_3_O_4_, Fe_5_C_2_ and acid sites), which cooperatively catalyse a tandem reaction. More significantly, the appropriate proximity of three types of active sites plays a crucial role in the successive and synergetic catalytic conversion of CO_2_ to gasoline. The multifunctional catalyst, exhibiting a remarkable stability for 1,000 h on stream, definitely has the potential to be a promising industrial catalyst for CO_2_ utilization to liquid fuels.

## Abstract (web summary)

Direct hydrogenation of CO_2_ into liquid fuels can mitigate CO_2_ emissions and reduce the rapid depletion of fossil fuels. Here, the authors show an iron-based multifunctional catalyst that converts CO_2_ to gasoline with high selectivity due to synergistic cooperation of multiple catalytic active sites.

For over 200 years, utilization of carbon-rich fossil fuels such as coal, oil and natural gas, has propelled the progress in human civilization, economic and social development^1^. However, the burning of fossil fuels gives rise to huge amounts of CO_2_ emissions, which brings about adverse climate changes. Converting CO_2_ from a detrimental greenhouse gas into value-added chemicals and fuels not only contributes to mitigating CO_2_ emissions, but also provides valuable fuels and thus enhances energy security in light of the depletion of fossil resources and the strong fluctuation of oil prices^1^,^2^,^3^,^4^. Unfortunately, the activation of CO_2_ and its hydrogenation to hydrocarbons or alcohols are challenging tasks because CO_2_ is a fully oxidized, thermodynamically stable and chemically inert molecule^5^. Another challenge arises with the low C/H ratio obtained during CO_2_ hydrogenation, due to the relatively low heat of CO_2_ adsorption on catalyst surface^6^. This favors the fast hydrogenation of surface-adsorbed intermediates, leading to the formation of methane and a decrease in chain growth^6^. Most research to date, not surprisingly, is focusing on the selective hydrogenation of CO_2_ to short-chain products, such as CO (refs 5, 7), CH_3_OH (refs 8, 9, 10), HCOOH (ref. 11), CH_4_ (ref. 12) and C_2_–C_4_ olefins^13^,^14^, while few studies on long-chain hydrocarbons^15^,^16^.

CO_2_ can be hydrogenated to hydrocarbons by either direct or indirect route. The direct CO_2_ hydrogenation (CO_2_-FT) is often described as the combination of the reduction of CO_2_ to CO via reverse water-gas shift (RWGS) reaction and subsequent hydrogenation of CO to hydrocarbons via Fischer–Tropsch synthesis (FTS)^6^. The indirect route is often performed by using different reactors with syngas (a mixture of CO and H_2_, derived from coal, natural gas and biomass) and/or methanol intermediate formation^17^. In contrast, the direct route is more economic and environmentally benign while this approach usually yields CO and light paraffins as major products owing to weak CO hydrogenation activity and over-hydrogenation of olefins^18^. Gasoline-range hydrocarbons are generally produced from refining of petroleum, or from syngas via FTS process, or from methanol-to-gasoline (MTG) process^19^. So far, there has been no report on highly selective synthesis of gasoline from direct CO_2_ hydrogenation. The key to this process is to search for a highly efficient catalyst.

Owing to their excellent ability to catalyse both RWGS and FTS processes and high olefinic nature of obtained products, iron-based catalysts remain the preferred catalyst candidate for CO_2_-FT process^2^. Furthermore, density functional theory calculations have demonstrated that Fe_3_O_4_(111) surface is very capable of activating CO_2_ (refs 20, 21). Typically, iron catalysts need alkali metal promotion to attain desired activity and selectivity. It was reported that the addition of Na is beneficial to olefin production^22^,^23^,^24^. The existence of Na obviously enhances the surface basicity and carburization of iron-based catalyst, making the catalyst very active for CO_2_ hydrogenation to light olefins^14^. Yet for the conventional iron-based catalysts, the hydrocarbon products generally follow the Anderson–Schulz–Flory (ASF) distribution, which is inherently wide and unselective^17^. More unfortunately, these hydrocarbons comprise mainly olefins and normal paraffins, with low octane number and unsuitable as gasoline fuel. Considering that zeolites are powerful in oligomerization/aromatization/isomerization of hydrocarbons due to their unique shape selectivity and acidity^17^, the combination of an iron-based CO_2_-FT catalyst with a zeolite into a multifunctional catalyst can shift product distribution towards high-octane gasoline-range isoparaffins and aromatics. In spite of considerable efforts made in the development of composite catalysts^15^,^18^, the selectivity to C_5+_ hydrocarbons, especially centred C_5_–C_11_ hydrocarbons, is not high enough owing to the poor coordination between the components of composite catalysts.

In present work, we report a high efficient multifunctional catalyst comprised of Na–Fe_3_O_4_ nanocatalyst and nanocrystalline HZSM-5 zeolite (Na–Fe_3_O_4_/HZSM-5 catalyst) for the direct conversion of CO_2_ to gasoline-range hydrocarbons. This catalyst displays record selectivity towards C_5_–C_11_ hydrocarbons (78%) as well as low CH_4_ and CO selectivity under industrially relevant conditions. It was also discovered that the choice of active components and the integration manner of the components are crucial to control the product selectivity.

## Results

### CO_2_ hydrogenation performance

**Figure 1:**
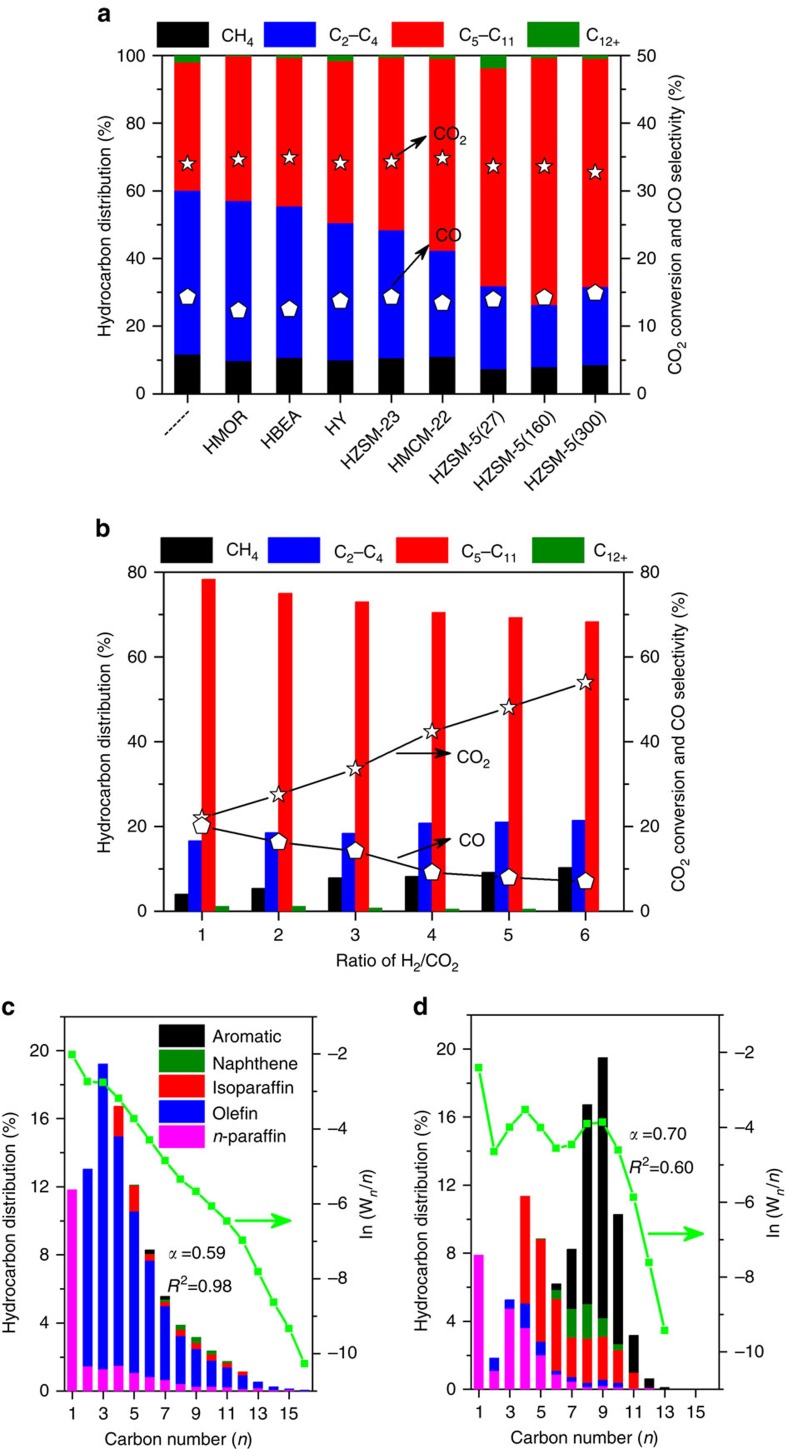
Catalytic performance for CO_2_ hydrogenation. (**a**) CO_2_ conversion and product selectivity over different Na–Fe_3_O_4_/Zeolite catalysts; reaction conditions: H_2_/CO_2_=3,320 °C, 3 MPa and 4,000 ml h^−1 ^g_cat_^−1^. (**b**) CO_2_ conversion and product selectivity at different H_2_/CO_2_ ratios over Na–Fe_3_O_4_/HZSM-5(160) catalyst at 320 °C, 3 MPa and 4,000 ml h^−1 ^g_cat_^−1^. (**c**,**d**) The detailed hydrocarbon product distribution obtained over Na–Fe_3_O_4_ (**c**) and Na–Fe_3_O_4_/HZSM-5(160) (**d**) catalysts, an additional ASF plot and *α* value comparison of above two catalysts are also depicted; *W*_*n*_ is the weight fraction of a product with *n* carbon atoms.

We initially prepared Na–Fe_3_O_4_ nanocatalyst by a simple one-pot synthesis method and then applied it to CO_2_ hydrogenation reaction. As shown in Fig. 1a, Na–Fe_3_O_4_ catalyst exhibited 12% selectivity to CH_4_, 38% selectivity to C_5_–C_11_ as well as a low CO selectivity (14%) at a CO_2_ conversion of 34%. Notably, the hydrocarbon distribution followed a fairly linear trend for Na–Fe_3_O_4_, implying an ASF product distribution (Fig. 1c). In our quest for a compatible zeolite, a series of zeolites like HY, HBEA, HMOR, HZSM-23, HMCM-22 and HZSM-5, possessing the ability to catalyse olefin oligomerization reaction in varying degrees, were coupled with Na–Fe_3_O_4_ catalyst for CO_2_ hydrogenation. The description of zeolite channels and NH_3_-TPD results were listed in Supplementary Table 1 and Supplementary Fig. 1. As shown in Fig. 1a, CO_2_ conversion and CO selectivity are not obviously related to zeolite type, predominantly decided by the first component of Na–Fe_3_O_4_, whereas the distribution of hydrocarbon products is evidently influenced by the zeolite pore structure on Na–Fe_3_O_4_/Zeolite catalysts for CO_2_ hydrogenation. It is noteworthy that three types of zeolites with 10 member ring (MR) channels exhibit higher C_5_–C_11_ selectivities in the order of HZSM-5 (3-dimensional)>HMCM-22 (2-dimensional)>HZSM-23 (1-dimensional). This result suggests zeolites with 10 MR channels can favour the oligomerization of olefins and the production of C_5_–C_11_ hydrocarbons. Besides the pore structure, the acidity, which is depended on the SiO_2_/Al_2_O_3_ ratio of zeolite, is another important factor affecting hydrocarbon distribution. It suggests that the stronger acidity of HZSM-5(27) could cause the over-cracking of heavy hydrocarbons to C_1_–C_4_ hydrocarbons, whereas the weaker one of the HZSM-5(300) is not beneficial to the oligomerization/isomerization/aromatization of primary CO_2_-FT products, thus both disfavour the selective production of C_5_–C_11_ hydrocarbons (Fig. 1a). In summary, HZSM-5(160) zeolite is suitable for C_5_–C_11_ hydrocarbon synthesis due to the presence of medium/strong acid sites and 3-dimensional pore structure.

The Na–Fe_3_O_4_/HZSM-5(160) multifunctional catalyst provided a CO_2_ conversion of 34% and selectivities to CH_4_, C_2_–C_4_, C_5_–C_11_ and C_12+_ hydrocarbons of 8, 18, 73 and 1%, respectively, under 320 °C, 3 MPa, and H_2_/CO_2_ ratio of 3 (Fig. 1a). Moreover, when the H_2_/CO_2_ ratio of feed gas was switched to 1, we observed an even higher selectivity to gasoline fraction (78%) and only 4% CH_4_ with a CO_2_ conversion of 22% over Na–Fe_3_O_4_/HZSM-5(160) catalyst (Fig. 1b). To our knowledge, this is the highest selectivity for gasoline-range hydrocarbons reported for CO_2_ hydrogenation (Supplementary Table 2). A higher H_2_/CO_2_ ratio benefits CO_2_ conversion, which rose to 54% at H_2_/CO_2_=6, for instance, whereas it disfavours the selective formation of gasoline fraction. Selectivities varied in the range from 68 to 78% for C_5_–C_11_ and 4 to 10% for CH_4_ in the investigated H_2_/CO_2_ ratio (1 to 6).

To further elucidate the function of HZSM-5, a detailed product distribution has been done on Na–Fe_3_O_4_/HZSM-5(160) catalyst (Fig. 1d). Compared with Na–Fe_3_O_4_ catalyst (Fig. 1c), the use of HZSM-5 as the second component significantly decreased the selectivities to CH_4_ and C_2_–C_4_, and altered the product distribution towards gasoline-range isoparaffins and aromatics. Moreover, oxygenates formation is inhibited at the presence of zeolite (Supplementary Table 3). An additional ASF plot and the probability of chain growth (*α*) value comparison of above two catalysts are also given in Fig. 1c,d. Relatively, Na–Fe_3_O_4_/HZSM-5 catalyst exhibited an *α* value of 0.70, higher than that of 0.59 for Na–Fe_3_O_4_ catalyst, confirming that the production of long-chain hydrocarbons was promoted on the multifunctional catalyst. The product distribution on the multifunctional catalyst deviated greatly from the typical ASF distribution, which could be attributed to the secondary reactions, such as oligomerization, isomerization and aromatization, occurring on zeolite acid sites.

Further, a tunable isoparaffin/aromatic ratio in gasoline-range hydrocarbons is achieved by simply altering zeolite type (Supplementary Fig. 2). Under the same conditions, HZSM-5(27), HZSM-5(160) and HZSM-5(300) with MFI topology produced higher amount of aromatics (up to 61% of aromatics in gasoline fraction) while HMCM-22 with MWW topology produced mainly isoparaffins (46% of isoparaffins in gasoline fraction). This phenomenon has a close correlation with the topology of different zeolites. HMCM-22 zeolite with 10 MR pore openings has a unique lamellar structure consisting of two independent pore systems, which leads to HMCM-22 with potential catalytic properties in isomerization, alkylation and disproportionation^25^. In addition, the major aromatics produced over Na–Fe_3_O_4_/HZSM-5 catalyst, were identified to be toluene, xylene, ethyltoluene, trimethylbenzene and dimethyl ethylbenzene, while less benzene and durene formed (both <1% in gasoline) (Supplementary Table 4). Such aromatic product distribution is evidently different from that derived from MTG process. It will not need an extra separation process usually applied in MTG process due to the higher content of durene in gasoline.

### Structural characterization

**Figure 2:**
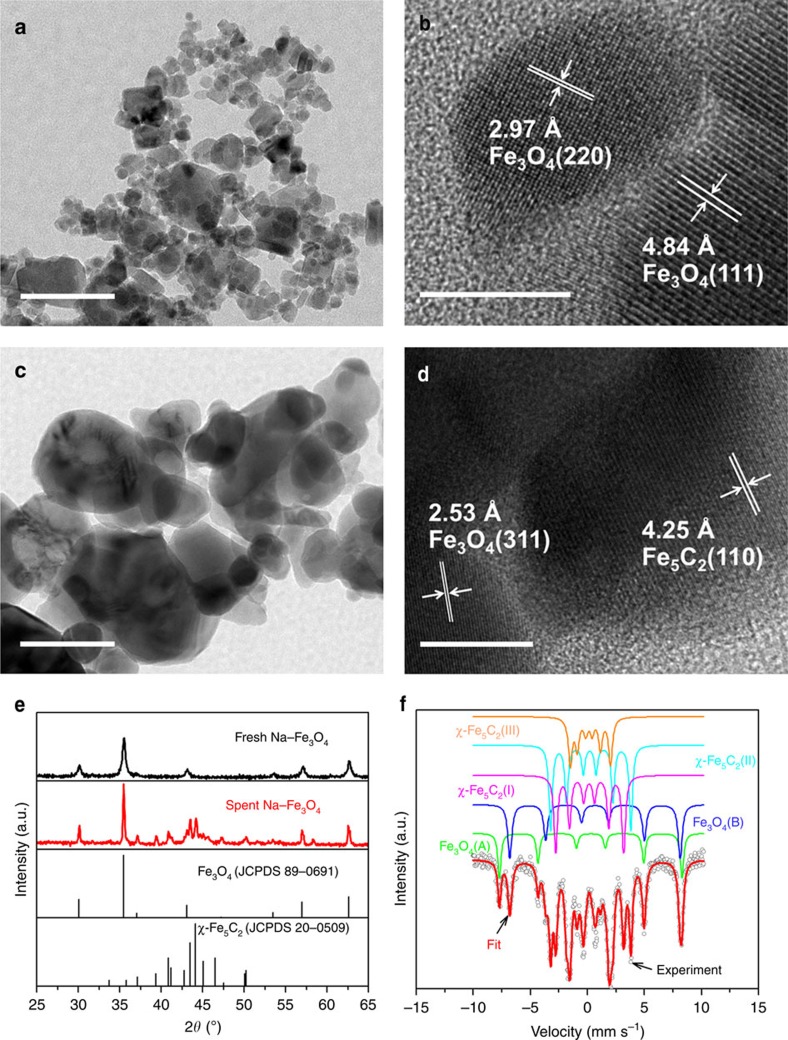
Structural characterization of Na–Fe_3_O_4_ catalyst. (**a**,**c**) TEM images of fresh (**a**) and spent (**c**) Na–Fe_3_O_4_ catalyst. Scale bar, 100 nm. (**b**,**d**) HRTEM images of fresh (**b**) and spent (**d**) Na–Fe_3_O_4_ catalyst. Scale bar, 10 nm. (**e**) XRD patterns of fresh and spent Na–Fe_3_O_4_ catalyst. (**f**) Mössbauer spectra of spent Na–Fe_3_O_4_ catalyst.

To reveal the nature of active sites that favors the formation of gasoline-range hydrocarbons, we resorted to multiple characterization techniques to investigate the structure of multifunctional catalyst. Na–Fe_3_O_4_ catalyst was composed of nanosized Fe_3_O_4_ with an average size of 13.1 nm, and the residual Na (0.7 wt%, determined by inductively coupled plasma (ICP)) was well distributed on the surface of Fe_3_O_4_ nanoparticles, with no obvious segregation (Fig. 2a,b,e; Supplementary Figs 3 and 4e). HZSM-5(160) was highly crystalline and appeared to be cuboid crystals ranged from 200 to 500 nm (Supplementary Fig. 4). Characterization of high resolution transmission electron microscopy (HRTEM), X-ray diffraction (XRD) and Mössbauer spectra showed that two different types of iron phase were discerned in the spent Na–Fe_3_O_4_ catalyst, with 32.4% of Fe_3_O_4_ and 67.6% of χ-Fe_5_C_2_ phase (Fig. 2c–f; Supplementary Table 5). Metallic iron is formed when Na–Fe_3_O_4_ is reduced in H_2_ prior to reaction (Supplementary Fig. 4c). Upon exposure of the catalyst to the reaction atmosphere, Fe_5_C_2_ and Fe_3_O_4_ are formed as a result of the interaction of metallic iron with carbon and oxygen species from the dissociated carbon oxides^26^. Appropriate proportion and arrangement of Fe_3_O_4_ (active sites for RWGS) and Fe_5_C_2_ (active sites for FTS)^26^, we speculated, is responsible for low CO selectivity (lower than 20%) with relatively high CO_2_ conversion during CO_2_ hydrogenation.

### Reaction scheme for CO_2_ hydrogenation

**Figure 3:**
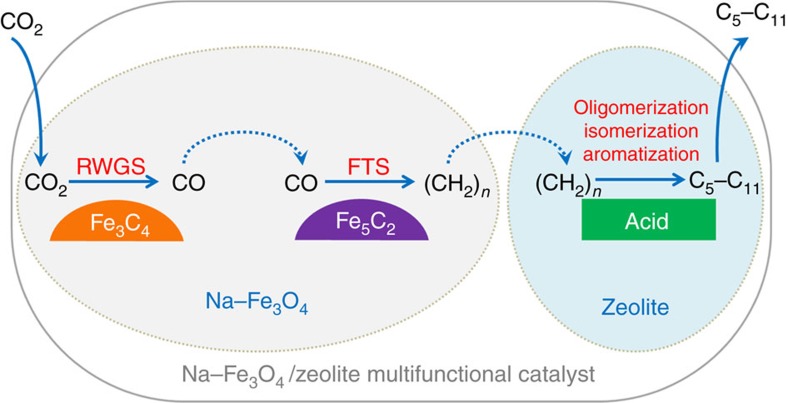
Reaction scheme for CO_2_ hydrogenation to gasoline-range hydrocarbons. The CO_2_ hydrogenation reaction over Na–Fe_3_O_4_/Zeolite multifunctional catalyst takes place in three steps: (1) an initially reduced to CO intermediate via RWGS, (2) a subsequent hydrogenation of CO to α-olefins intermediate via FTS and (3) the formation of gasoline-range hydrocarbons via the acid-catalysed oligomerization, isomerization and aromatization reactions.

In the basis of the results above, we propose a reaction scheme of CO_2_ hydrogenation to hydrocarbons over Na–Fe_3_O_4_/Zeolite multifunctional catalyst as illustrated in Fig. 3. This scheme indicates that the multifunctional catalyst, with three types of active sites, exhibits complementary and compatible properties. During CO_2_ hydrogenation, CO_2_ is initially reduced to CO by H_2_ via RWGS on Fe_3_O_4_ sites, followed by a subsequent hydrogenation of CO to α-olefins via FTS on Fe_5_C_2_ sites. The olefin intermediates generated on the iron-based catalyst then diffuse to zeolite acid sites, on which they undergo acid-catalysed reactions (oligomerization, isomerization and aromatization), as a consequence, the gasoline-range isoparaffins and aromatics are selectively formed and finally diffuse out of zeolite pores. Besides, CO_2_ conversion and product selectivity could be modulated by varying the mass ratio of Na–Fe_3_O_4_ relative to zeolite (Supplementary Fig. 5), which provides further support to the above hypothesis that Na–Fe_3_O_4_/Zeolite catalyst is multifunctional and the reaction involves intermediate migration among different active sites.

### Proximity effect in multifunctional catalysts

**Figure 4:**
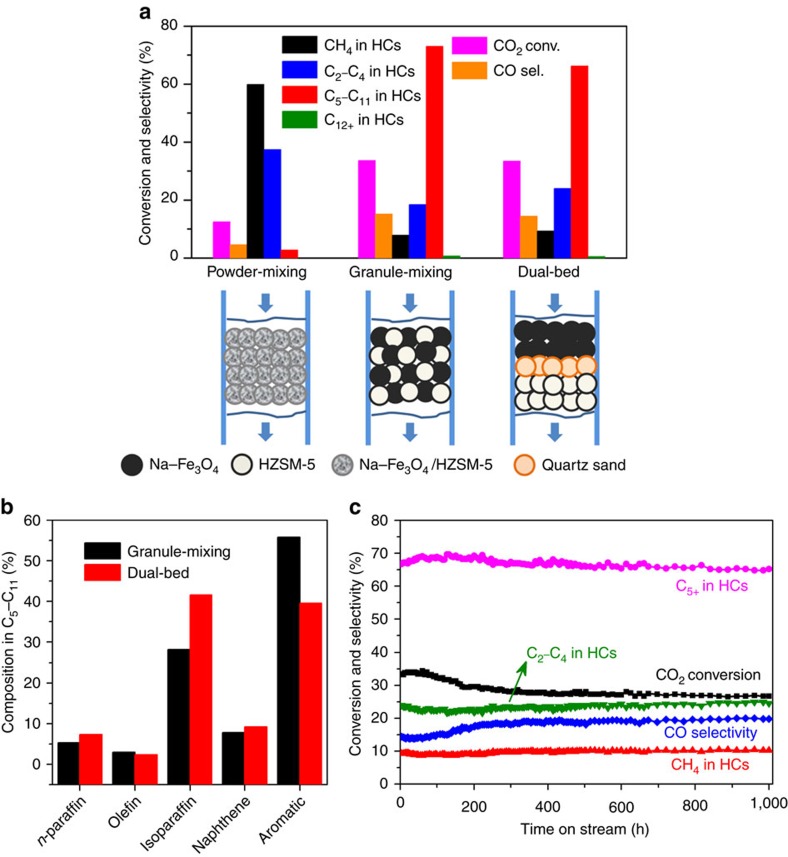
CO_2_ hydrogenation performance over the multifunctional catalysts with different proximity. (**a**) CO_2_ conversion and product selectivity over different combinations of Na–Fe_3_O_4_ and HZSM-5 catalysts conducted at the same reaction conditions as Fig. 1a; HCs: hydrocarbons. (**b**) The composition of gasoline-range hydrocarbons on different Na–Fe_3_O_4_/HZSM-5(160) composite catalysts. (**c**) The stability of the Na–Fe_3_O_4_/HZSM-5 catalyst with dual-bed configuration under the same reaction conditions as Fig. 1a. The hydrocarbon selectivities are normalized with the exception of CO.

The proximity of the two components in multifunctional catalysts has been reported to exert significant influence on catalytic activity (refs 27, 28, 29). In our case, we found that it is also vital for selective conversion of CO_2_ to hydrocarbons (Fig. 4a). When Na–Fe_3_O_4_ and HZSM-5 were integrated by powder mixing, the closest proximity between iron-based sites and zeolite acid sites turned out to be detrimental, exhibiting a very low CO_2_ conversion (13%) and high undesired CH_4_ selectivity up to 60%. The reason, we speculated, is that the zeolite acid sites poison the Na-induced alkali sites on the Fe_3_O_4_ surface, leading to a decrease in the surface basicity and carburization degree of Fe_3_O_4_ catalyst. Likewise, another 2%Na–10%Fe/HZSM-5 catalyst with a close intimacy we prepared by an incipient wetness impregnation method as a comparison also presented a poor performance on CO_2_ hydrogenation (Supplementary Table 3). When Na–Fe_3_O_4_ and HZSM-5 were combined by granule mixing, the distance between iron-based and zeolite acid sites was enlarged, and the olefin intermediates formed on iron-based sites diffused through wide pores to zeolite, where they immediately underwent oligomerization, isomerization and aromatization reactions, giving rise to the highest C_5_–C_11_ selectivity (73%) at a CO_2_ conversion of 34%. It demonstrated an appropriate distance between iron-based and acid sites is critical for achieving excellent performance. With regard to dual-bed configuration, where HZSM-5 was packed below Na–Fe_3_O_4_ and separated by a thin layer of inert quartz sand, the distance between iron-based and acid sites got larger. It exhibited a slightly lower C_5_–C_11_ selectivity (67%) and the same CO_2_ conversion as the manner of granule mixing.

Note that the composition of gasoline-range hydrocarbons also relies on different combinations of Na–Fe_3_O_4_ and HZSM-5 catalysts (Fig. 4b). It is inclined to produce more aromatics under the manner of granule mixing, while more isoparaffins are produced under the dual-bed configuration. We have further measured the stability of the Na–Fe_3_O_4_/HZSM-5 catalyst with dual-bed configuration (Fig. 4c). It demonstrated good stability over 1,000 h on stream. The C_5+_ selectivity stably maintained at 67±2% throughout the test. CO_2_ conversion was only reduced by 6% within the first 300 h on stream, related to the loss of active Fe surface as a result of the sintering of iron taken place during this stage (Fig. 2c)^30^. Afterwards, the iron species tended to be stable and thus CO_2_ conversion was constant during the next few hundred hours. The total coke deposit on HZSM-5 was only 3.7 wt% (Supplementary Fig. 6). Thus, the present multifunctional catalyst is stable and suitable for the direct conversion of CO_2_ to gasoline.

## Discussion

In conclusion, we have succeeded in preparing a highly selective Na–Fe_3_O_4_/HZSM-5 multifunctional catalyst for the direct production of gasoline from CO_2_ hydrogenation. This catalyst enables RWGS over Fe_3_O_4_ sites, olefin synthesis over Fe_5_C_2_ sites, and oligomerization/aromatization/isomerization over zeolite acid sites. The concerted action of the active sites calls for precise control of their structures and proximity. It exhibited 78% selectivity to C_5_–C_11_ as well as low CH_4_ and CO selectivity, and gasoline fraction are mainly isoparaffins and aromatics thus favoring the octane number. Moreover, the composition of C_5_–C_11_ can be tuned by the choice of zeolite type and the integration manner of multifunctional catalyst. In particular, this multifunctional catalyst and the process may allow use of the feed gas with a low H_2_/CO_2_ ratio thus reduce the cost of hydrogen. This study paves a new path for the synthesis of liquid fuels by utilizing CO_2_ and H_2_. Furthermore, it provides an important approach for dealing with the intermittency of renewable sources (sun, wind and so on) by storing energy in liquid fuels.

## Methods

### Catalyst preparation

We prepared the Na–Fe_3_O_4_ nanocatalyst by a one-pot synthesis method. Typically, 31.62 g FeCl_3_·6H_2_O and 12.54 g of FeCl_2_·4H_2_O were added to 150 ml deionized (DI) water containing 5.1 ml of 12.1 mol l^−1^ HCl with stirring to form a clear solution. In the above solution, 1.5 mol l^−1^ of NaOH was then added dropwise under stirring at 60 °C. Consequently, an instant black precipitate was generated, and the pH value of final suspension was maintained at 10. The resulting suspension was kept with stirring for 1 h. The product was separated by a magnet, and washed once with 800 ml of DI water to obtain a Fe_3_O_4_ nanocatalyst modified with a certain content of residual Na. The fresh-made nanocatalyst was dried overnight at 60 °C and directly used for CO_2_ hydrogenation reaction without further thermal treatment to maintain their nanostructure and morphology. The chemical reaction for synthesis of Fe_3_O_4_ is given by equation (1)^31^.


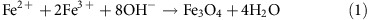


In the above synthesis, we prepared and modified the catalysts simultaneously without any extra steps. NaOH is served as not only the precipitating agent but also the promoter source. By changing the number of washing times and the volume of water consumption for each wash, the content of promoter can be regulated easily. For comparison, a Fe_3_O_4_ nanocatalyst without Na modification was prepared by the same method just substituting NH_3_·H_2_O (5 wt%) for NaOH (1.5 mol l^−1^) as the precipitating agent.

HY (SiO_2_/Al_2_O_3_=5), HMCM-22 (SiO_2_/Al_2_O_3_=30) and HZSM-5 zeolites (SiO_2_/Al_2_O_3_=27, 160, 300) were commercially available from Nankai University catalyst company, China. HBEA (SiO_2_/Al_2_O_3_=25) and HMOR (SiO_2_/Al_2_O_3_=20) were purchased from Zeolyst International. HZSM-23 (SiO_2_/Al_2_O_3_=80) was synthesized by a hydrothermal method^32^. Before used, the zeolites were calcined in air at 500 °C for 4 h.

The Na–Fe_3_O_4_/Zeolite catalyst was typically prepared by granule mixing Na–Fe_3_O_4_ catalyst with zeolite at a mass ratio of the two components of 1:1 unless otherwise noted. Take the preparation of Na–Fe_3_O_4_/HZSM-5 catalyst with granule mixing as an example. Na–Fe_3_O_4_ and HZSM-5 were pressed into pellets (30 MPa), crushed and sieved to 20–40 meshes (granule sizes of 380–830 μm), respectively. Then, the granules of the two samples were mixed together by shaking in a vessel. For preparation of Na–Fe_3_O_4_/HZSM-5 catalyst with powder mixing (Fig. 4a), Na–Fe_3_O_4_ and HZSM-5 were mixed in an agate mortar for 2 min, and then pressed, crushed and sieved to 20–40 meshes. The obtained sample was denoted as Na–Fe_3_O_4_/HZSM-5-PM.

For comparison, 2 wt% Na–10 wt% Fe/HZSM-5(160) catalyst was prepared by incipient wetness impregnation with aqueous Fe(NO_3_)_3_ and NaNO_3_ in addition. After impregnation for 12 h, the samples were dried at 60 °C for 8 h and calcined at 500 °C for 4 h.

### Catalyst characterization

The Na content of the Na–Fe_3_O_4_ nanocatalyst was analysed with an inductively coupled plasma optical emission spectrometer (ICP-OES, Perkin-Elmer Optima 7300DV) after the catalyst sample had been digested with hydrochloric acid at room temperature. XRD spectra of the powder catalysts were recorded with a PANalytical X’Pert Pro diffractometer using Cu-Kα (40 kV, 40 mA) irradiation. For the XRD test of the reduced catalysts, passivation treatment with 1% O_2_/99% N_2_ at room temperature was conducted after reduced at 350 °C for 8 h in H_2_. The specific surface area of the catalysts was analysed by the BET method carrying out N_2_ adsorption measurements at −196 °C on a Quantachrome instrument. All the samples were degassed at 300 °C for 6 h under vacuum before adsorption.

The morphology of the catalysts was characterized by scanning electron microscopy (SEM) on a JSM-7800F microscope operated at an accelerating voltage of 1.5 kV. Transmission electron microscopy (TEM) images were obtained on a JEM-2100 system (JEOL) with an acceleration voltage of 200 kV. The samples were ultrasonically suspended in ethanol and placed onto a carbon film supported over a Cu grid for that purpose.

NH_3_ temperature-programmed desorption (NH_3_-TPD) measurements were performed on a home-made setup. Typically, 100 mg sample was loaded into a U-shaped quartz microreactor (i.d.=4 mm) and pretreated at 600 °C for 0.5 h in flowing He. After pretreatment, the sample was cooled down to 100 °C and saturated with NH_3_. The sample was flushed in He flow for 0.5 h to remove the gas phase NH_3_. Then, NH_3_-TPD was carried out in a constant flow of He (30 ml min^−1^) from 100 to 700 °C at a heating rate of 10 °C min^−1^. The concentration of NH_3_ in the exit gas was continuously detected by a gas chromatograph (SHIMADZU) with a thermal conductivity detector (TCD).

Thermo-gravimetric and differential thermal analyses (TG–DTA) of zeolite samples were performed on a Perkin-Elmer Diamond TGS-2 and DTA1700 apparatus. The experiments were carried out in the temperature range of 30–1,000 °C, with a heating rate of 10 °C min^−1^ in flowing air (40 ml min^−1^).

The ^57^Fe Mössbauer spectra (MES) of the catalysts were carried out on a Topologic 500A spectrometer driving with a proportional counter at room temperature. The radioactive source was ^57^Co (Rh) moving in a constant acceleration mode. Data analyses were performed assuming a Lorentzian lineshape for computer folding and fitting. The components of iron phases were identified based on their Mössbauer parameters including isomer shift, quadruple splitting and magnetic hyperfine field.

### Catalytic performance tests

CO_2_ hydrogenation reactions were performed in a stainless steel fixed-bed reactor with an inner diameter of 14 mm. Typically 1 g of composite catalyst (20–40 meshes) with Na–Fe_3_O_4_/Zeolite=1/1 (mass ratio) was used unless otherwise stated. Prior to reaction, the catalyst was in-situ reduced at 350 °C for 8 h in a pure H_2_ flow at atmospheric pressure. After reduction, the reactor was cooled to 320 °C. Then the reactant gas mixture H_2_/CO_2_/N_2_ (containing 4 vol% N_2_ as the internal standard) was fed into the reactor, and the system was pressured gradually to 3 MPa. All of the products from the reactor were introduced in a gaseous state and analysed with two online gas chromatographs (GC) (VARIAN 3800). N_2_, CO, CO_2_ and CH_4_ were analysed using a GC system equipped with a TCD, a Hayesep C column and a molecular sieve 13X column. The organic compounds including hydrocarbons and oxygenates were analysed using another GC system equipped with a flame ionization detector (FID) and a PONA capillary column. The reaction was carried out under the conditions of H_2_/CO_2_=3,320 °C, 3 MPa and 4,000 ml h^−1^ g_cat_^−1^ unless otherwise stated. For a test with Na–Fe_3_O_4_ only of Fig. 1a, 0.5 g of catalyst was used, and the flow rate of feed gas is 4,000 ml h^−1^. Moreover, the hydrocarbon distribution was calculated based on the total carbon moles with a unit of C-mol% on all tested catalysts. The carbon balances of various reactions were calculated, which were over 95% for all reactions. The catalytic performances after at least 2 h on stream were typically used for discussion.

CO_2_ conversion was calculated by equation (2):


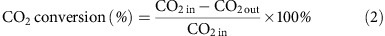


where CO_2 in_ and CO_2 out_ represent the molar fraction of CO_2_ at the inlet and outlet, respectively.

CO selectivity was calculated according to equation (3):


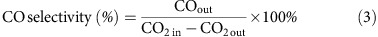


where CO_out_ represents the molar fraction of CO at the outlet.

The selectivity of different hydrocarbon in total hydrocarbons was given as equation (4):


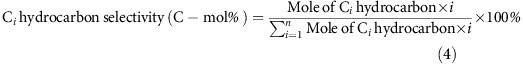


### Data availability

The data supporting the findings of this study are available within the article and its Supplementary Information files. All other relevant source data are available from the corresponding author upon reasonable request.

## Additional information

**How to cite this article:** Wei, J *et al*. Directly converting CO_2_ into a gasoline fuel. *Nat. Commun.* **8**, 15174 doi: 10.1038/ncomms15174 (2017).

**Publisher’s note:** Springer Nature remains neutral with regard to jurisdictional claims in published maps and institutional affiliations.

## Supplementary Material

Supplementary Information Supplementary Figures, Supplementary Tables and Supplementary References
