# Erratum: Directly converting CO_2_ into a gasoline fuel

**DOI:** 10.1038/ncomms16170

**Published:** 2017-10-12

**Authors:** Jian Wei, Qingjie Ge, Ruwei Yao, Zhiyong Wen, Chuanyan Fang, Lisheng Guo, Hengyong Xu, Jian Sun

Nature Communications 8: Article number: 15174 ; DOI: 10.1038/ncomms15174 (2017); Published 05 02 2017; Updated 10 12 2017

This Article contains an error in Fig. 3; in that, ‘Fe_3_C_4_’ in the orange semi-ellipse should be ‘Fe_3_O_4_’.

