# Supplementary material for: Directly converting CO2 into a gasoline fuel
Source: Nat Commun. 2017 May 2;8:15174. doi: 10.1038/ncomms15174 (PMC5418575; doi:10.1038/ncomms15174)
Supplement: Supplementary Information — Supplementary Figures, Supplementary Tables and Supplementary References [file ncomms15174-s1.pdf]

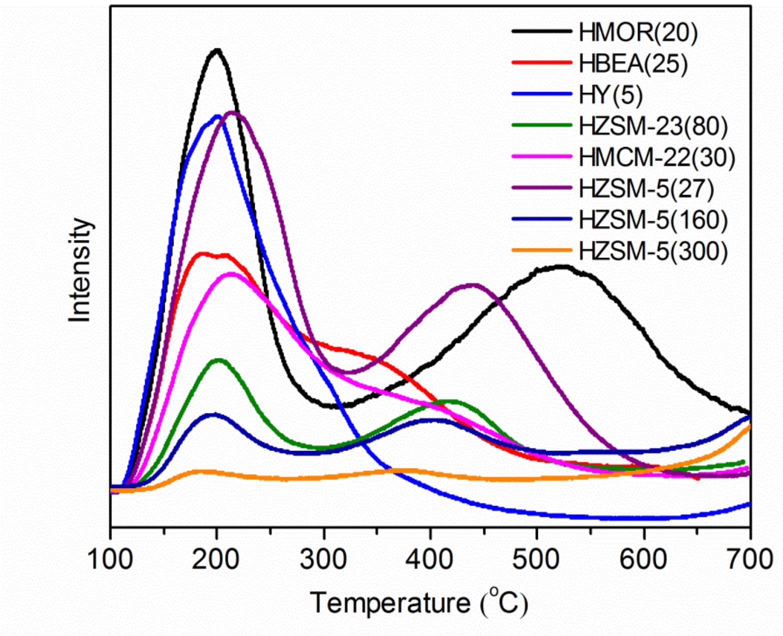

Supplementary Figure 1 | NH_3-TPD profiles of different zeolites.

The acidity of the zeolites was investigated by ammonia temperature-programmed desorption (NH_3-TPD) studies. The NH_3-TPD spectra mainly show one peak at 110–300 °C and one peak above 310 °C, which correspond to the weak acid sites and strong acid sites of the zeolites, respectively.

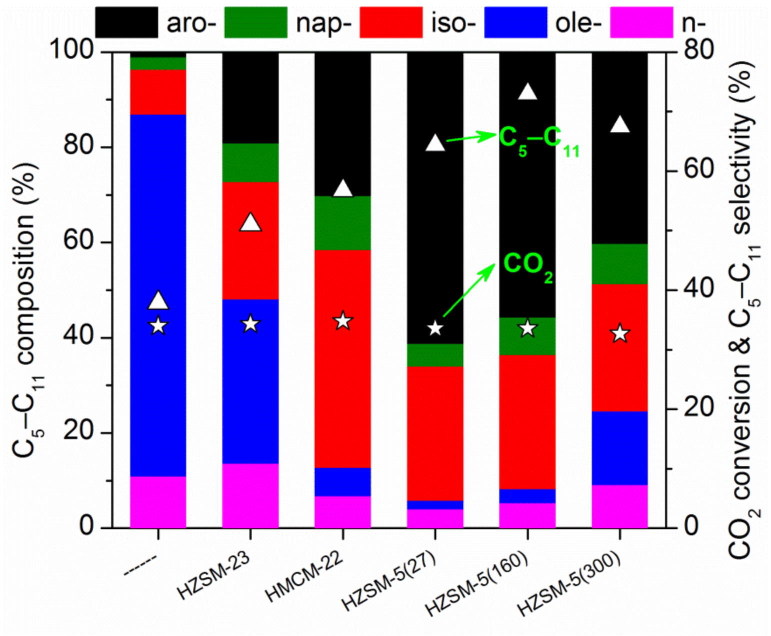

Supplementary Figure 2 | The C_5–C_{11} composition over different Na–Fe_3O_4/Zeolite multifunctional catalysts. Note: n-: n-paraffin; ole-: olefin; iso-: isoparaffin; nap-: naphthene; aro-: aromatic.

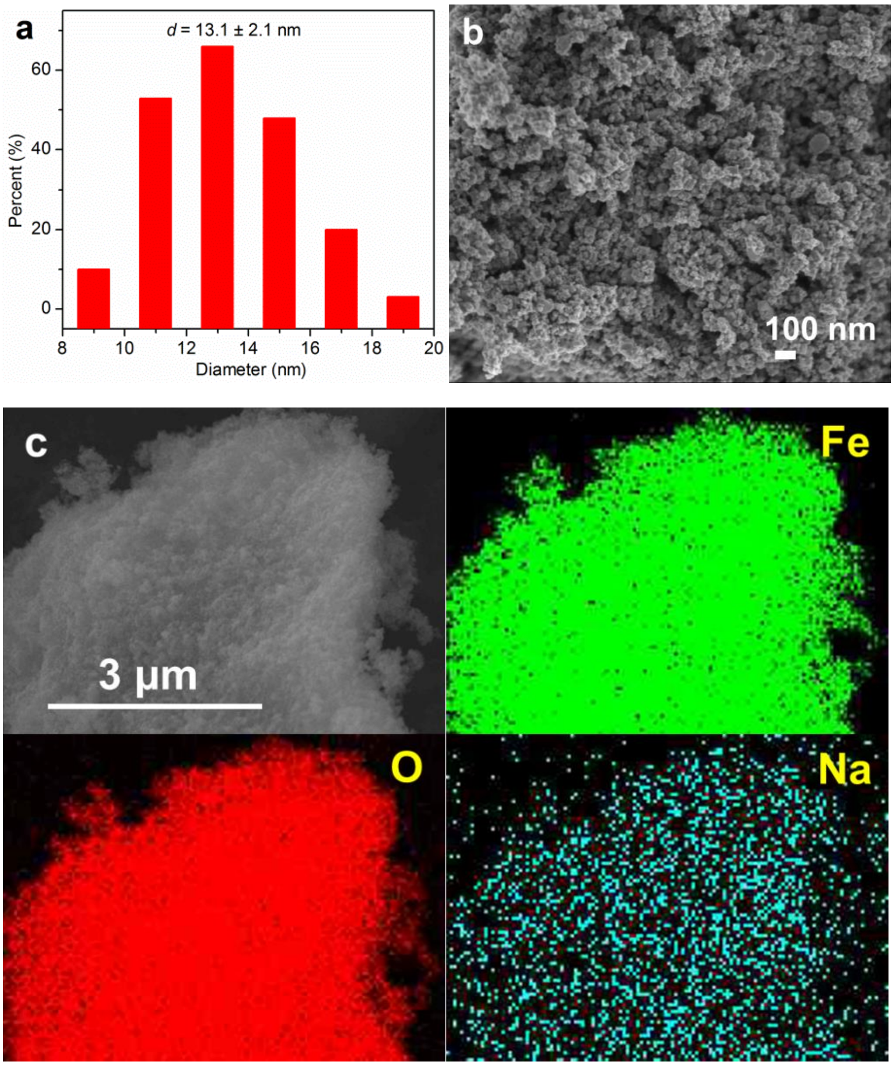

Supplementary Figure 3 | Characterization of Na–Fe_3O_4 catalyst. **a**, Particle size distribution of Na–Fe_3O_4 catalyst (calculated on the basis of 200 nanospheres from TEM images). **b**, SEM images of Na–Fe_3O_4 catalyst. **c**, SEM micrograph and corresponding EDS elemental mapping of Na–Fe_3O_4 catalyst. Iron, oxygen and sodium elements are colored in green, red and cyan, respectively.

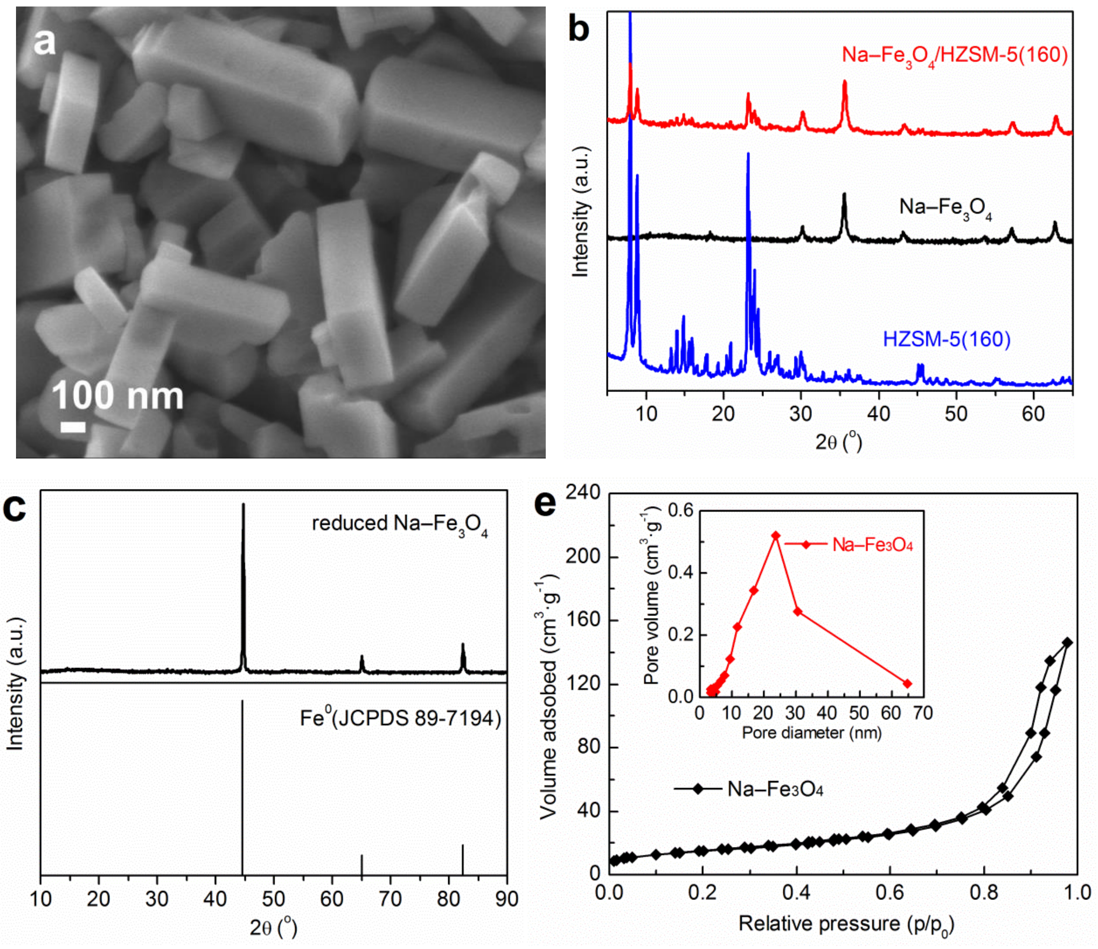

d

Catalyst	BET surface area ($m^2\ g^{-1}$)	External surface area ($m^2\ g^{-1}$)	Micropore surface area ($m^2\ g^{-1}$)	Micropore volume ($cm^3\ g^{-1}$)	Pore volume ($cm^3\ g^{-1}$)	Average mesopore diameter (nm)
Fe_3O_4	57.8	57.8	0.0	0.0	0.27	18.8
Na–Fe_3O_4	52.2	52.2	0.0	0.0	0.23	17.4
HZSM-5(160)	444.8	54.9	389.9	0.16	0.23	2.5

Supplementary Figure 4 | SEM, XRD and BET analyses of the catalysts. a, SEM images of HZSM-5(160). **b**, XRD patterns of Na–Fe_3O_4, HZSM-5(160) and the resulting Na–Fe_3O_4/HZSM-5 composite catalyst. The XRD of HZSM-5 shows peaks corresponding to hydrogen aluminum silicate hydrate, as expected. **c**, XRD pattern of reduced Na–Fe_3O_4. **d**, BET analyses of Fe_3O_4, Na–Fe_3O_4 and HZSM-5(160) catalysts. **e**, N_2 adsorption–desorption isotherm and pore distribution of the Na–Fe_3O_4 catalyst. The catalyst exhibits a very broad pore distribution in the range of 7–40 nm with a maximum at ~22 nm, mainly formed by the agglomerates of Fe_3O_4 nanoparticles.

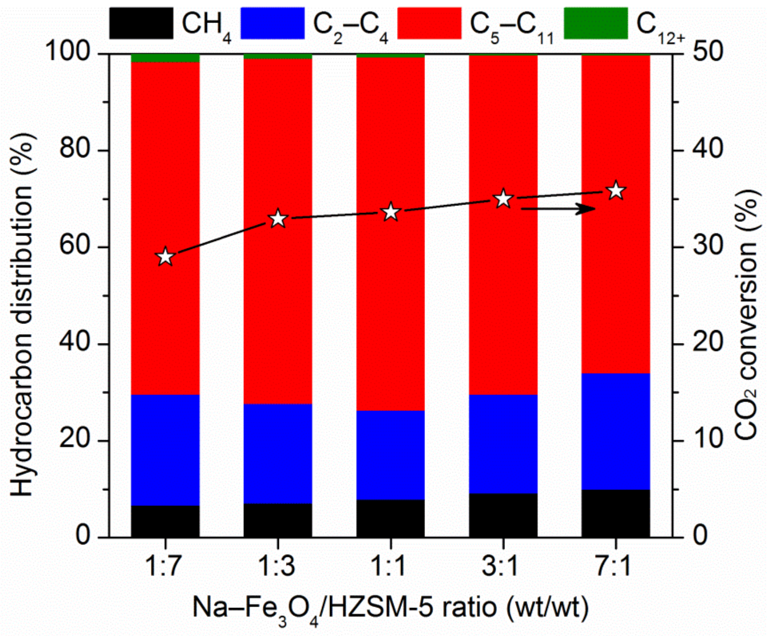

Supplementary Figure 5 | Catalytic performances over composite catalysts as a function of the mass ratio of Na–Fe_3O_4/HZSM-5 in the composite catalysts. Reaction conditions: H_2/CO_2 = 3.0, 320 ^{o}C, 3.0 MPa, 4,000 ml h^{-1} g_{cat}^{-1}.

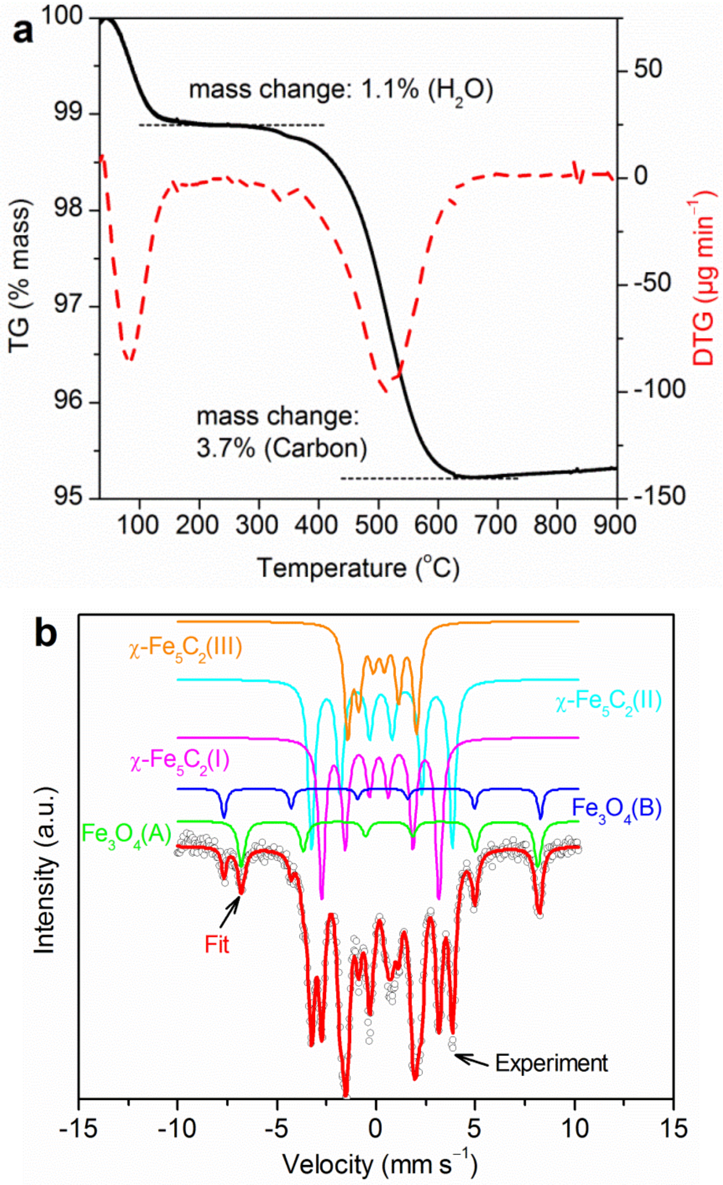

Supplementary Figure 6 | TG and Mössbauer spectra analyses of the catalysts. a, TG & DTG of HZSM-5 in the composite catalyst after reaction tests of 1,000 h in Fig. 4c. **b**, Mössbauer spectra of Na–Fe_3O_4 in the composite catalyst after reaction tests of 1,000 h in Fig. 4c. The detailed Mössbauer parameters are listed in Supplementary Table 5. ^{57}Fe Mössbauer spectroscopy is a powerful tool to identify and quantify the iron phases formed during CO_2 hydrogenation reaction. As shown in Supplementary Table 5, the sextets with Hhf of 495 and 463 kOe can be attributed to tetrahedral site (A site) and octahedral site (B site) of magnetite (Fe_3O_4), respectively[1]. The three

sextets with Hhf values of 183 (Sextet I), 220 (Sextet II) and 108 kOe (Sextet III), respectively, agree well with the literature values for three sites in Hägg iron carbide (χ-Fe_5C_2)[2].

Supplementary Table 1 | Description of the channel systems of zeolites [a].

Zeolite type	Framework type	Ring size (T-atoms)	Channel system	Channel system
HMOR	MOR	12,8,5,4	[001] **12** 6.5 × 7.0* ↔ [001] **8** 2.6 × 5.7**	1-dimensional
HBEA	BEA	12,6,5,4	<100> **12** 6.6 × 6.7** ↔ [001] **12** 5.6 × 5.6*	3-dimensional
HY	FAU	12,6,4	<111> **12** 7.4 × 7.4***	3-dimensional
HZSM-23	MTT	10,6,5	[001] **10** 4.5 × 5.2*	1-dimensional
HMCM-22	MWW	10,6,5,4	[001] **10** 4.0 × 5.5** | [001] **10** 4.1 × 5.1**	2-dimensional
HZSM-5	MFI	10,6,5,4	{[100] **10** 5.1 × 5.5 ↔ [010] **10** 5.3 × 5.6}***	3-dimensional

[a] Data are from the International Zeolite Association (IZA).

Supplementary Table 2 | CO_2 hydrogenation performance data in our work and from other literatures.

Catalyst	T (^oC)	P (bar)	CO_2 conv. (%)	CO sel. (%)	Hydrocarbon distribution (%)		
					CH_4	C_2–C_4	C_{5+}
Fe-Zn-Zr/HZSM-5 [3]	360	50	19.5	57.4	2	46	52
Fe-Zn-Zr@HZSM-5–HY [4]	340	50	14.2	40.5	1.8	64.2	34.0
Fe-Ce/KY [5]	300	10	20.1	34.6	8.9	41.5	49.6
Fe/RbY [6]	300	10	17.2	31.6	9.5	36.1	54.4
Fe-Cu-Na/HZSM-5 [7]	250	20	12.3	19.6	28.5	42.3	29.2
$CuFeO_2$ [8]	300	10	17.3	31.7	2.7	31.0	66.3 [a]
Cu-Zn-Al/HB [9]	300	9.8	27.6	53.4	1.5	93.5	5.0
Fe-Zn-Zr/HY [10]	340	50	22.4	50.4	1.8	83.9	14.3
Cu-Zn-Zr-Al/Pdβ [11]	260	20	25.2	47.2	1.2	77.6	21.2
**Na–Fe_3O_4/HZSM-5** [b]	320	30	22.0	20.1	4.0	16.6	**79.4**

[a] The C_{5+} products cover the gasoline (C_5–C_{11}), diesel (C_{12}–C_{21}) ranges, and ~15% of waxy hydrocarbons (C_{25+}).

[b] The data in our work. The C_{5+} products cover 78.3% of C_5–C_{11}, and 1.1% of C_{12+} hydrocarbons.

Supplementary Table 3 | Reaction performance for CO_2 hydrogenation [a].

Catalyst	CO_2 conv. (%)	Product selectivity (%)			Hydrocarbon distribution (C-mol %)			
		CO	Oxy-	HC	CH_4	$C_{2–4}$	$C_{5–11}$	C_{12+}
Fe_3O_4	21.4	10.6	1.6	87.8	53.3	41.8	4.9	0.0
Na–Fe_3O_4	34.0	14.3	4.0	81.7	11.7	48.4	37.9	2.0
Na–Fe_3O_4/HZSM-5(160)	33.6	14.2	0.0	85.8	7.9	18.4	73.0	0.7
2%Na–10%Fe/HZSM-5	5.4	29.5	0.0	70.5	49.3	43.9	6.8	0.0

[a] Reaction conditions: H_2/CO_2 = 3.0, 320 ^oC, 3.0 MPa, 4,000 ml h^{-1}.

Supplementary Table 4 | Detailed composition of the aromatics fraction over Na–Fe_3O_4/HZSM-5(160) catalysts in Fig. 1a,d.

Aromatics		Content in all aromatics (C-mol %)
C_6	benzene	0.9
C_7	toluene	8.5
C_8	ethylbenzene	3.1
	xylene (m-, p-, o-)	25.3
C_9	propylbenzene	0.8
	ethyltoluene (m-, p-, o-)	22.8
	trimethylbenzene	13.4
C_{10}	diethylbenzene	2.9
	methyl propylbenzene	2.4
	dimethyl ethylbenzene	11.9
	durene (tetramethylbenzene)	1.2
C_{11}	----	5.3
C_{12+}	----	1.5
SUM		100.0

Supplementary Table 5 | Detailed Mössbauer parameters.

Figure	Assignment	Mössbauer parameters			
		IS (mm/s)	QS (mm/s)	Hhf (kOe)	Spectral contribution (%)
Fig. 2f	Fe_3O_4 (A)	0.30	–0.02	496	13.2
	Fe_3O_4 (B)	0.67	0.00	463	19.2
	χ-Fe_5C_2 (I)	0.18	0.05	185	25.0
	χ-Fe_5C_2 (II)	0.25	0.09	218	25.9
	χ-Fe_5C_2 (III)	0.20	0.13	109	16.7
Supplementary Fig. 6b	Fe_3O_4 (A)	0.31	–0.03	495	4.1
	Fe_3O_4 (B)	0.67	0.02	463	9.0
	χ-Fe_5C_2 (I)	0.17	0.07	183	31.2
	χ-Fe_5C_2 (II)	0.27	0.05	220	32.8
	χ-Fe_5C_2 (III)	0.21	0.16	108	22.9

Supplementary References

1. Berry, F. J., Skinner, S. & Thomas, M. F. Mössbauer spectroscopic examination of a single crystal of Fe_3O_4. *J. Phys.: Condens. Matter* **10**, 215-220 (1998).
2. Raupp, G. B. & Delgass, W. N. Mössbauer investigation of supported Fe and FeNi catalysts: II. Carbides formed Fischer-Tropsch synthesis. *J. Catal.* **58**, 348-360 (1979).
3. Tan, Y., Fujiwara, M., Ando, H., Xu, Q. & Souma, Y. Syntheses of isobutane and branched higher hydrocarbons from carbon dioxide and hydrogen over composite catalysts. *Ind. Eng. Chem. Res.* **38**, 3225-3229 (1999).
4. Wang, X. *et al.* Synthesis of isoalkanes over core (Fe-Zn-Zr)-shell (zeolite) catalyst from CO_2 hydrogenation. *Chem. Commun.* **52**, 7352-7355 (2016).
5. Nam, S.-S., Kishan, G., Lee, M.-W., Choi, M.-J. & Lee, K.-W. Selective synthesis of C_2–C_4 olefins and C_{5+} hydrocarbons over unpromoted and cerium-promoted iron catalysts supported on ion exchanged (H, K) zeolite-Y. *J. Chem. Res.(S)*, 344-345 (1999).
6. Nam, S.-S., Kim, H., Kishan, G., Choi, M.-J. & Lee, K.-W. Catalytic conversion of carbon dioxide into hydrocarbons over iron supported on alkali ion-exchanged Y-zeolite catalysts. *Appl. Catal. A: Gen* **179**, 155-163 (1999).
7. Xu, Q. *et al.* Hydrogenation of carbon dioxide over Fe–Cu–Na/zeolite composite catalysts: Na migration via solid–solid reaction and its effects on the catalytic activity. *J. Mol. Catal. A: Chem.* **136**, 161-168 (1998).
8. Choi, Y. H. *et al.* Carbon dioxide Fischer-Tropsch synthesis: a new path to

carbon-neutral fuels. *Appl. Catal. B: Environ* **202**, 605-610 (2017).

9. Fujiwara, M., Satake, T., Shiokawa, K. & Sakurai, H. CO_2 hydrogenation for C_{2+} hydrocarbon synthesis over composite catalyst using surface modified HB zeolite. *Appl. Catal. B: Environ* **179**, 37-43 (2015).

10. Bai, R., Tan, Y. & Han, Y. Study on the carbon dioxide hydrogenation to iso-alkanes over Fe–Zn–M/zeolite composite catalysts. *Fuel Process. Technol.* **86**, 293-301 (2004).

11. Li, C., Yuan, X. & Fujimoto, K. Direct synthesis of LPG from carbon dioxide over hybrid catalysts comprising modified methanol synthesis catalyst and β-type zeolite. *Appl. Catal. A: Gen* **475**, 155-160 (2014).
